# Fitness consequences of altered feeding behavior in immune-challenged mosquitoes

**DOI:** 10.1186/s13071-016-1392-x

**Published:** 2016-02-29

**Authors:** Johanna R. Ohm, Janet Teeple, William A. Nelson, Matthew B. Thomas, Andrew F. Read, Lauren J. Cator

**Affiliations:** Center for Infectious Disease Dynamics, Departments of Biology and Entomology, Pennsylvania State University, University Park, PA USA; Center for Infectious Disease Dynamics, Department of Entomology, Pennsylvania State University, University Park, PA USA; Department of Biology, Queens University, Kingston, Ontario Canada; Grand Challenges in Ecosystems and Environment, Department of Life Sciences, Imperial College, Silwood Park Campus, London, UK

**Keywords:** *Anopheles*, Sickness behavior, Fitness, Parasite manipulation, Malaria

## Abstract

**Background:**

Malaria-infected mosquitoes have been reported to be more likely to take a blood meal when parasites are infectious than when non-infectious. This change in feeding behavior increases the likelihood of malaria transmission, and has been considered an example of parasite manipulation of host behavior. However, immune challenge with heat-killed *Escherichia coli* induces the same behavior, suggesting that altered feeding behavior may be driven by adaptive responses of hosts to cope with an immune response, rather than by parasite-specific factors. Here we tested the alternative hypothesis that down-regulated feeding behavior prior to infectiousness is a mosquito adaptation that increases fitness during infection.

**Methods:**

We measured the impact of immune challenge and blood feeding on the fitness of individual mosquitoes. After an initial blood meal, *Anopheles stephensi* Liston mosquitoes were experimentally challenged with heat-killed *E. coli* at a dose known to mimic the same temporal changes in mosquito feeding behavior as active malaria infection. We then tracked daily egg production and survivorship of females maintained on blood-feeding regimes that either mimicked down-regulated feeding behaviors observed during early malaria infection, or were fed on a four-day feeding cycle typically associated with uninfected mosquitoes.

**Results:**

Restricting access to blood meals enhanced mosquito survival but lowered lifetime reproduction. Immune-challenge did not impact either fitness component. Combining fecundity and survival to estimate the population-scale intrinsic rate of increase (*r*), we found that, contrary to the mosquito adaptation hypothesis, mosquito fitness decreased if blood feeding was delayed following an immune challenge.

**Conclusions:**

Our data provide no support for the idea that malaria-induced suppression of blood feeding is an adaptation by mosquitoes to reduce the impact of immune challenge. Alternatively, the behavioral alterations may be neither host nor parasite adaptations, but rather a consequence of constraints imposed on feeding by activation of the mosquito immune response, i.e. non-adaptive illness-induced anorexia. Future work incorporating field conditions and different immune challenges could further clarify the effect of altered feeding on mosquito and parasite fitness.

**Electronic supplementary material:**

The online version of this article (doi:10.1186/s13071-016-1392-x) contains supplementary material, which is available to authorized users.

## Background

The feeding behavior of mosquitoes is altered after infection with malaria parasites [1–10]. Blood feeding, a behavior with high risk of mosquito mortality, has been reported to be suppressed during the non-infectious stage of parasite development and enhanced during the infectious stage [7]. These behavioral changes likely increase the probability of onward transmission and hence parasite fitness [8, 9], leading to the assumption that altered mosquito behavior is a classic case of parasite manipulation [6]. However, the behavior is not specific to malaria parasites as immune challenge with heat-killed *E. coli* can induce the same behavioral changes [5], suggesting that mosquito behavioral changes may be a sickness behavior arising from a general immune response rather than malaria-specific manipulation [11, 12].

Restricted feeding during infection is a common sickness behavior observed in numerous host-parasite systems [13–15] and is hypothesized to be adaptive in other insects [16, 17]. Altered feeding behavior in malaria-infected mosquitoes could similarly have evolved as an adaptation to enhance the fitness of infected mosquitoes if feeding during infection has a cost. We propose that the altered feeding behavior of anophelines during malaria infection is another example of adaptive illness-induced anorexia, and explains the same phenomenon previously attributed to parasite-manipulation [6]. If the altered behaviors are adaptive, then we expect that mosquitoes feeding on these altered regimes should have increased fitness following an immune-challenge compared to those that continue to feed normally.

At first glance, delayed blood feeding seems likely to reduce fitness because mosquitoes require a blood meal to mature eggs. However, infection and blood feeding can decrease mosquito survivorship [18–20] and, in the days after immune-challenge, mosquitoes produce fewer eggs and have reduced egg and larval viability [21–25]. Egg production in anophelines has been shown to trade-off with innate immune responses via a shared pathway involved in nutrient transport for reproduction and immune systems [26], and may also be mediated by shared pathways involved in insulin signaling [27]. In other species of mosquitoes, survival-reproduction trade-offs have been used to explain enhanced longevity during infection with malaria parasites, e.g. reduced egg lays in infected *Culex* mosquitoes was correlated with enhanced longevity [28]. Avoiding blood meals soon after challenge may, therefore, be adaptive if this behavior helps reduce the fitness costs of infection. The costs of delayed reproduction could be ameliorated by benefits from extended survival or compensatory reproduction that may occur once infection is controlled. Compensatory reproduction occurs in other iteroparous systems, including other invertebrates (e.g. [29, 30]) and illness-induced anorexia can increase resistance or tolerance to infection in other insects [17, 31–33]. The situation remains unclear in anophelines because experiments to date have focused on just the first or second clutches after immune challenge (an exception is found in [22] which found fecundity reductions for malaria-infected mosquitoes across three gonotrophic cycles), rather than looking at total lifetime metrics. Additionally, previous studies exploring the effects of diet on mosquito longevity, reproduction and fitness have shown mixed results, making it unclear whether classic understandings of survival benefits to chronic restriction diets apply in the anopheline system [34, 35].

Here, we tested the hypothesis that avoiding blood feeding during the period following an immune-challenge is adaptive (fitness-enhancing) for mosquito-hosts. We tested the effects of delayed blood feeding, immune challenge, and their interaction on the fitness of the malaria vector *Anopheles stephensi* Liston by administering immune challenges to mosquitoes on feeding regimes that either mimicked the delayed feeding behaviors of malaria-infected mosquitoes (restricted access to blood meals) or were fed blood meals on regular four-day intervals mimicking typical feeding of uninfected mosquitoes (unaltered access to blood meals)*.* We employed a life table design to capture two components of fitness, an individual’s daily fecundity and survival for mosquitoes in all treatment groups. Both components were then incorporated into models that estimated the intrinsic rate of increase (*r*) of mosquitoes that followed the experimental feeding regimes with and without an initial immune challenge. We expected that delayed blood feeding, which we refer to here as restricted, would be costly to mosquitoes because of reduced reproductive opportunities. Here we address whether the relative costs are lessened for mosquitoes undergoing an immune challenge. If our hypothesis is correct, we expect that the fitness costs of delayed blood feeding would be reduced for mosquitoes that were immune-challenged as compared to those that were not administered an immune-challenge. Understanding how known changes in blood feeding behaviors during infection impact mosquito life history traits and fitness is informative for understanding why these behaviors may have evolved, and for predicting epidemiological patterns of vector-borne disease.

## Methods

### Mosquito rearing

Eggs from over 1000 females of *Anopheles stephensi* (Penn State maintained colony, sourced from Walter Reed Army Institute of Research) were placed in plastic trays (25 x 25 x 7 cm) filled with 1 L distilled water and maintained at 27 °C and 80 % relative humidity. Upon reaching second instar, larvae were transferred to fresh trays at a density of 400 larvae/L. Larvae were fed 5 mL ground fish flakes per day (TetraFin, Melle, Germany) suspended in water at a concentration of 10 mg/L in each tray. Pupae were collected and placed in cages for emergence. Adults were provided with 10 % glucose solution, supplemented with 0.05 percent paraminobenzoic acid (PABA). Females were housed with males for 2-5 days prior to the initial blood meal to provide the opportunity for mating. One thousand adult females were offered an initial blood meal from one of ten uninfected C57BL/6 mice and 400 blood-fed females were randomly selected to be individually tracked throughout the remainder of the experiment. The experiment was repeated for two replicates, with the second replicate having an additional immune-challenge sham group (details below) and hence a larger total sample size of 600 blood-fed mosquitoes. The study was carried out in accordance with the recommendations in the guide for the Care and Use of Laboratory Animals of the National Institutes of Health. The protocol was approved by the Animal Care and Use Committee of the Pennsylvania State University (#44512).

### Immune-challenge and feeding treatments

**Fig. 1:**
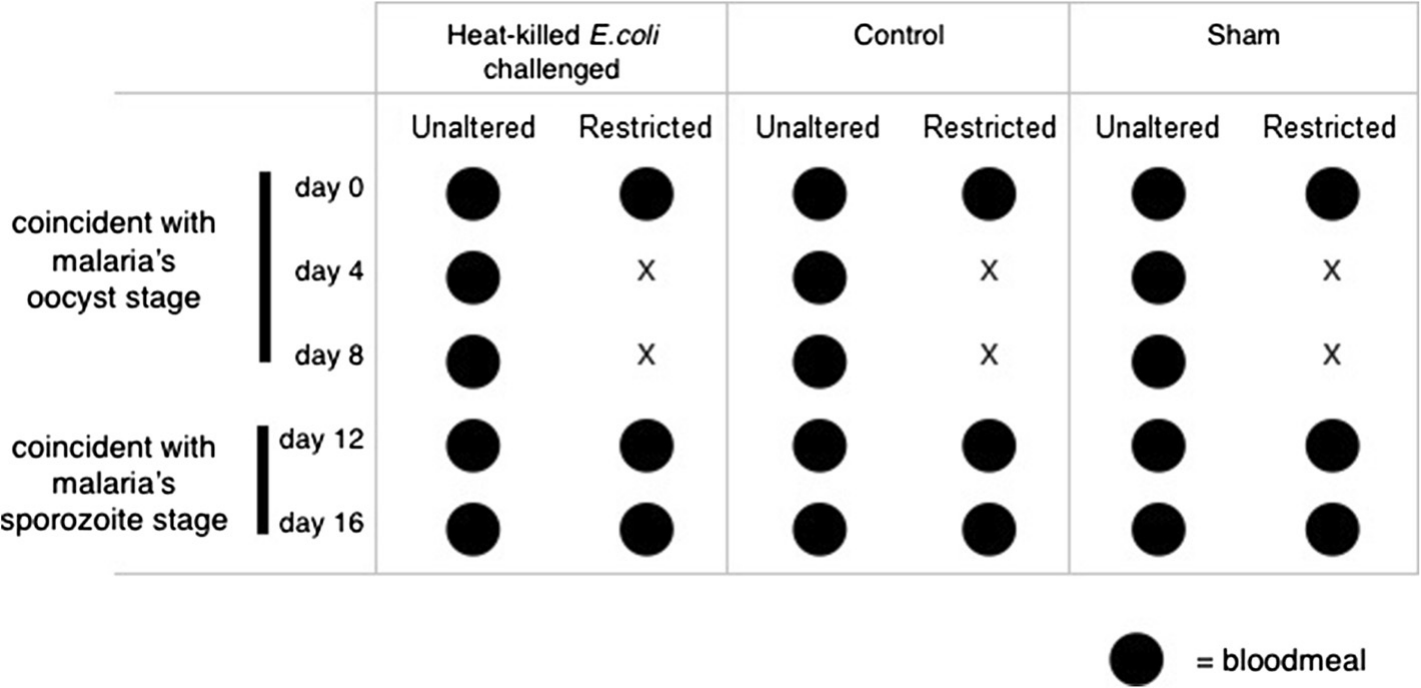
Experimental design for feeding regimes under different immune challenges. Filled black circles denote times when blood meals were offered. Immune-challenge treatments are shown in columns. Each immune-challenge treatment column is split to show feeding patterns for the two feeding regimes used in our experiment: restricted and unaltered. All females were fed on experimental days 0, 12 and 16. Restricted feeding regimes mimicked altered feeding behaviors observed during malaria infection and were denied blood meals on days 4 and 8. Unaltered feeding regimes fed every 4 days. A sample size of 100 female mosquitoes was used for each treatment in each of two replicates. We included a sham treatment only in Replicate 2. All mosquitoes received a 2.5 percent sucrose solution on days without blood meals

Individual mosquitoes were offered an uninfected blood meal on Day 0 (Fig. 1). Females that fed on Day 0 were subjected to one of three immune-challenge treatments: (1) cold anesthesia and an injection with 200,000 heat-killed *E. coli* (immune-challenge), (2) cold anesthesia and an injection with sterile LB broth which served as a control for mechanical damage (sham, experimental replicate 2 only), or (3) cold anesthesia alone (control). This dose of heat-killed *E.coli* (200,0000) has been found to stimulate the mosquito immune response [36], and alter feeding behavior in a way that mimics active malaria infection [5].

Following the immune treatment (immune-challenge, sham or control), females were placed alone in clear 50 ml plastic tubes covered with white mesh. Approximately 5 ml of fresh water filled the base of tubes to prevent desiccation and provide oviposition sites. Mosquitoes were then randomly assigned into one of two feeding regimes. The unaltered feeding regime groups were offered blood meals on days 4, 8, 12 and 16 after the initial feed, which mimics a feeding schedule corresponding to the average gonotrophic cycle in mosquitoes with unaltered behavior (Fig. 1). The restricted feeding regime groups were offered blood meals on days 12 and 16 after the initial feed, mimicking the feeding behavior of mosquitoes with down-regulated feeding response after malaria infection (Fig. 1). These blood meals came from 20 uninfected female C57BL/6 mice. Blood meals were offered at a distance of ~10 cm, ensuring that immune-challenged mosquitoes would feed. Previous studies have shown that the altered behavior we were testing is a dampened propensity to respond to host cues at distances of 48 cm or more [5].

Individual mosquitoes were tracked daily to check for survival and egg laying. Female mosquitoes that laid eggs were removed from their tubes and assigned a fresh tube with fresh water. This allowed us to separate eggs into distinct clutches. Eggs were collected and allowed to hatch to determine egg batch viability from half of all individuals that reproduced. Egg batch viability was measured as the percent of eggs that hatched. Hatching occurred within 24 h after eggs were laid.

Between blood meals, all females were provided with a cotton ball soaked in 2.5 % sucrose solution, a concentration lower than the concentration used for colony maintenance to induce a degree of nutritional stress likely closer to field conditions. Cotton balls were removed 10 to 12 h before blood meals.

### Life-table analysis of fitness effects

We used the life table data to explore the significance of an interaction between immune-challenge treatment and feeding regime. We analyzed survival and reproduction components of fitness separately, and then analyzed the cumulative impact on relative fitness using intrinsic rate of increase (*r*) (Additional file 1).

Mosquito survival was analyzed using a Cox proportional hazard model on lifespan [37–40]. Model covariates included our immune-challenge treatment, meal treatment, wing length, and experimental replicate. Mosquito reproduction was analyzed first by considering the proportion of individuals reproducing across treatments using a quasi-binomial generalized linear model. The fecundity of individuals that reproduced was then analyzed using a generalized linear model assuming a quasi-poisson distribution, which allows for over dispersion. The analyses were done using the glm function in the R statistical environment [40].

For all response variables related to fecundity (proportion reproducing, clutch size, total lifetime reproduction), we compared the fit of models with increasing complexity using the corrected Quasi-Akaike Information criteria (QAIC_c_) [41–43]. Like AIC_c_, QAIC_c_, identifies the candidate model from a suite of potential models that best fits the data. Use of Quasi-Akaike Information criteria is appropriate when the data for a response variable are overdispersed [42, 43]. Full models included feeding regime, immune challenge treatment, replicate, winglength and all interactions. Final models were selected by comparing QAIC_c_ values. The model with the lowest QAIC_c_ value was chosen as the best-fit model. When multiple models performed equally well (within ΔQAIC_c_ <2) the least complex model was chosen as the best fit. We report test statistics from these best-fit selected models using analysis of variance F-tests. F-test results for all pairwise comparisons were consistent with the results of our analysis using information criterion.

Fitness was calculated as the per-capita intrinsic rate of increase (*r*) for each individual life history, which combines the effects of age-specific fecundity and survivorship. Specifically, the observed life-table data were used to parameterize a population model whose growth rate is a measure of fitness (*r*) under unlimited resources (details below). It was assumed that all individuals in the population model followed the observed life-table data, which means that the per-capita growth rate (*r*) was a measure of fitness for that individual’s life-history strategy.

Fitness calculations were generated using a continuous time model developed to match the life-history of mosquitoes in this experimental setting. Full development of the model, details of the analysis, and evaluation of assumptions are presented in Additional file 1, a brief synopsis of which is presented below. The life-table data recorded for each individual includes the day of death (*α*) and number of eggs laid each day (*b*_*i*_ for day *i* since maturation). To track the population growth rate that would emerge from many individuals following a specific set of life-table values, the model needed to account for egg (*E*(*t*)), larval (*L*(*t*)) and adult (*A*_*i*_(*t*)) stages, where the index *i* denotes the time since maturation in adults. The population model is

$$ \begin{array}{l}\frac{dE(t)}{dt}\kern0.5em =\kern0.5em {\displaystyle \sum_{i=\mathsf{0}}^{\alpha }{b}_i{A}_i(t)-}{\displaystyle \sum_{i=\mathsf{0}}^{\alpha }{b}_i{A}_i\left({t}_1\right){S}_E\mathit{\hbox{-}}{\delta}_EE}\\ {}\frac{dL(t)}{dt}\kern0.5em =\kern0.5em {\displaystyle \sum_{i=\mathsf{0}}^{\alpha }{b}_i{A}_i\left({t}_1\right){S}_E-}{\displaystyle \sum_{i=\mathsf{0}}^{\alpha }{b}_i{A}_i\left({t}_2\right){S}_E{S}_L\mathit{\hbox{-}}{\delta}_LL}\\ {}\frac{d{A}_j(t)}{dt}\kern0.5em =\kern0.5em {\displaystyle \sum_{i=\mathsf{0}}^{\alpha }{b}_i\Big({A}_i\left({t}_{\mathsf{2}}-j\right)-}{A}_i\left({t}_{\mathsf{2}}-j-1\right)\Big){S}_E{S}_L\\ {}\kern2.5em {S}_E\kern0.5em =\kern0.5em  \exp \left(-{\delta}_E{\tau}_E\right)\\ {}\kern2.5em {S}_L\kern0.5em =\kern0.5em  \exp \left(-{\delta}_L{\tau}_L\right)\\ {}\kern3em {t}_1=\kern0.5em t-{\tau}_E\\ {}\kern3em {t}_{\mathsf{2}}=\kern0.5em t-{\tau}_E-{\tau}_L\end{array} $$

where *τ*_*E*_ and *τ*_*L*_ are the egg and larvae stage durations respectively, *δ*_*E*_ and *δ*_*L*_ are the stage-specific mortality rates, and *S*_*E*_ and *S*_*L*_ are through-stage survivorship. The observed life-table data for adult longevity (*α*) and birth rate (*b*_*i*_) are explicit parameters in the model, and the asymptotic growth rate provides an estimate of fitness (*r*) for each individual.

The effect of treatment on fitness was analyzed using the same approach as fecundity and survivorship. Specifically, we used a generalized linear model to evaluate the effect of the treatments on fitness (*r*). The fit models were strongly under-dispersed (lower variance than expected, c-hat < 1), and evaluation of the mean-variance relationship suggested the data are gamma distributed. As in the fecundity analyses, the fit of models were compared to our fitness measure (*r*) with increasing complexity using AIC_c_. AIC_c_ was utilized here because intrinsic rates of increase data did not need to be corrected for overdispersion. QAIC_c_ and AIC_c_ are identical when c-hat = 1 [42, 43].

To quantify the impact of any fitness reductions observed in our continuous time model on rates of evolution, the time to *quasi-loss* (*T*), which is the length of time it would take a phenotype with the altered (restricted) feeding behavior of delaying blood meals after the immune-challenge to be reduced to 1 percent relative abundance by a phenotype with the unaltered feeding behavior, was calculated. Assuming the phenotypes start at equal relative abundance, the expression for quasi-loss is

$$ T\kern0.5em =\kern0.5em \frac{ \ln \left({\scriptscriptstyle \frac{1\hbox{-} 0.01}{0.01}}\right)}{s} $$

where *s* is the selection coefficient. The selection coefficient is calculated as the difference between the mean fitness values of mosquitoes displaying different phenotypes. All models and statistical tests were done in the R software environment [40].

For survival analysis, and models and analysis of fitness (*r*), mosquitoes that died of unnatural causes (handling errors) or failed to comply with the assigned feeding regime were excluded (<10 percent of individuals). Low compliance was expected in challenged mosquitoes on the unaltered feeding treatment, as our hypothesis was based on these mosquitoes having suppressed feeding behavior. However compliance was comparable between groups (Replicate 1: Wald *χ*2 = 1.4, df = 3 for days when compliance was not 100 percent for all groups, p > 0.5; Replicate 2: Wald *χ*2 = 26.96, df = 17, p > 0.05, compliance only significantly different for controls on day 4, see Additional file 2: Table S1), likely because blood meals were offered at a short host-range on anesthetized hosts that did not require active host-seeking or maneuvering around host defenses. The behavioral phenotype we were exploring, suppressed host seeking behavior that was described by long-range assays in [5], did not extend to suppressed feeding at the short host-range of <10 cm used in our design.

For analysis of reproduction, the same mosquitoes that did not comply or died of unnatural causes (handling errors) were also excluded from the analysis of total lifetime reproduction (N = 55), but not excluded from clutch-level analyses. Unnatural death or noncompliance would result in lower total lifetime reproduction unrelated to treatment.

Finally, in experimental replicate 2, no differences were noted between the sham and control treatment groups for any of the response variables (Additional file 2: Tables S2-S4), so data on the individuals in the sham groups were omitted to be conservative.

## Results

### The effect of immune-challenge and feeding regime on survival

**Fig. 2:**
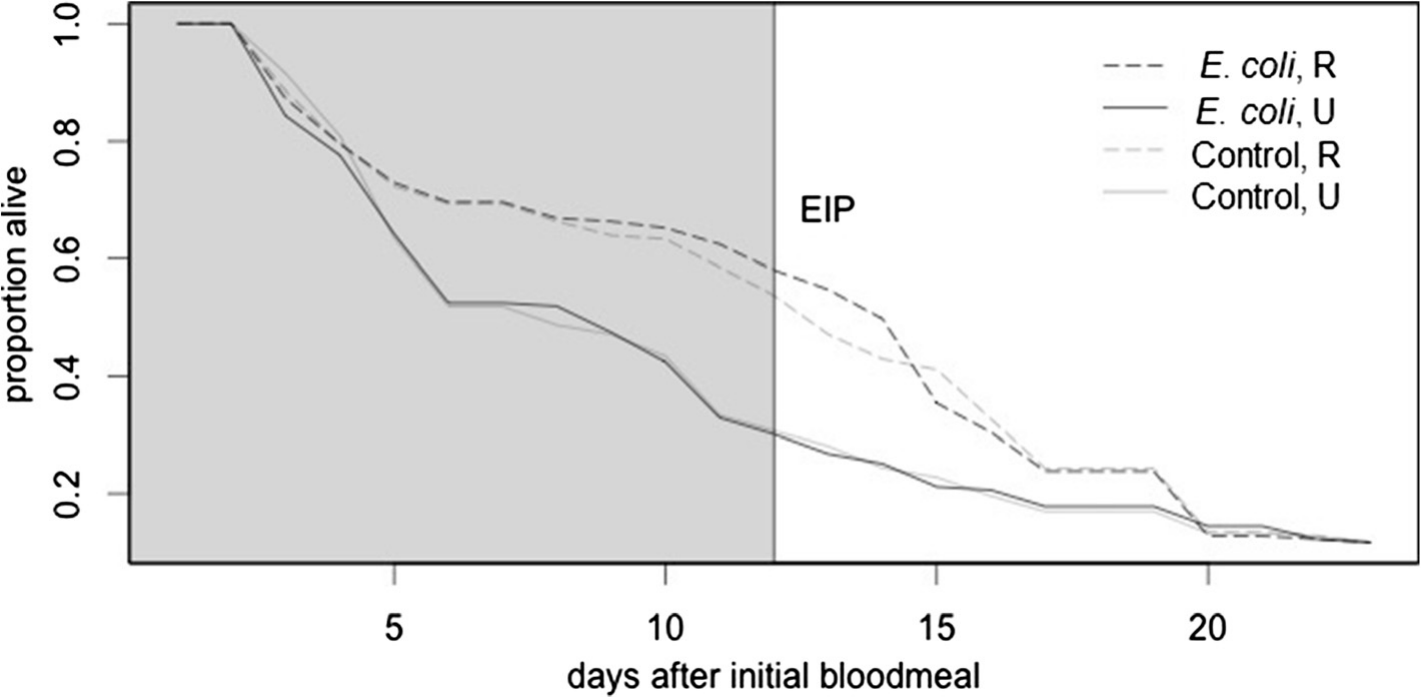
Mosquito survival varies by feeding regime. Mosquitoes that were offered fewer blood meals experienced extended survival, compared to mosquitoes that fed more frequently on the unaltered ‘U’ regime. The y-axis is the proportion of mosquitoes in each treatment alive for each day following the initial blood meal, with day after the initial blood meal shown on the x-axis. Dashed lines represent restricted ‘R’ feeding regimes and solid lines represent unaltered ‘U’ feeding regimes. Controls and heat-killed *E. coli* challenged mosquitoes are shown in gray and black, respectively. The vertical black line indicates the approximate extrinsic incubation period for *P. falciparum* at 27 °C [44–47]

Females in the restricted blood meal treatment had higher daily survival probabilities than females that fed on an unaltered, four-day cycle (Wald *χ*2 = 22.94, df = 1, p < 0.0001, Fig. 2). Immune-challenge did not affect survival of female mosquitoes (Wald *χ*2 = 5.26, df = 2, p > 0.05, Fig. 2). There was no significant interaction between immune-challenge and feeding treatment on survival (Wald *χ*2 = 28.66, df = 5, p > 0.5).

When survivorship was compared at day 12 after the immune challenge, the time period corresponding to the extrinsic incubation period for *P. falciparum* at 27 °C [44–47], we found that restricting blood meals increased the probability that mosquitoes would survive long enough to transmit the parasite (Wald *χ*2 = 32.29, df = 5, p < 0.05), while immune-challenge had no effect on survival over this time period (p > 0.05) (Fig. 2). Approximately twice the number of mosquitoes survived beyond day 12 when on a restricted feeding regime compared to an unaltered feeding regime (Fig. 2).

### The effect of immune-challenge and feeding regime on fecundity

The majority of the mosquitoes that blood-fed subsequently laid eggs (Table 1). Immune challenge with heat-killed *E. coli* reduced the proportion of mosquitoes that produced egg batches at least once during the lifespan of the unaltered feeding regimes, independent of any potential differences in mortality, but feeding regime alone had no effect (Table 1; model selection based on QAIC_c_ values in the Additional file 2: Table S5). Neither feeding regime nor an interaction between feeding regime and immune-challenge were predictive of proportion of mosquitoes reproducing (Additional file 2: Table S5).

**Table 1:** Mosquito reproduction and fitness

Clutch	Mean clutch size					
	Control, U (189)	Control, R (185)	Heat-killed *E. coli*, U (183)	Heat-killed *E. coli*, R (188)	Sham, U (100)	Sham, R (75)
1	124 ± 3 (114)	118 ± 4 (106)	109 ± 4 (86)	109 ± 11 (111)	119 ± 4 (56)	123 ± 6 (41)
2	117 ± 6 (53)	N/A	117 ± 4 (63)	N/A	108 ± 5 (35)	N/A
3	111 ± 8 (37)	N/A	107 ± 6 (32)	N/A	105 ± 8 (23)	N/A
4	121 ± 6 (27)	103 ± 5 (41)	108 ± 5 (23)	113 ± 6 (39)	127 ± 8 (15)	103 ± 5 (22)
5	101 ± 9 (13)	108 ± 7 (18)	91 ± 5 (12)	101 ± 10 (22)	107 ± 8 (11)	103 ± 6 (15)
Proportion reproducing	0.66	0.62	0.55	0.61	0.60	0.60
Lifetime total reproduction (LTR)	236 ± 16 (110)	173 ± 10 (104)	237 ± 17 (90)	169 ± 11 (108)	287 ± 26 (45)	220 ± 16 (38)
Fitness (*r*)	0.197 ± .002	0.186 ± .002	0.192 ± .002	0.183 ± .003	0.199 ± .002	0.190 ± .003

Mean clutch size per blood meal, i.e. number of eggs laid, ± standard error, per gonotrophic cycle, for females in each group surviving to oviposition. Numbers in parentheses represent the number of mosquitoes that reproduced (eggs > 0), and were thus included in the calculation. Unaltered ‘U’ feeding regimes were fed every 4 days, while restricted ‘R’ feeding regimes fed on days 0, 12, and 16 after immune-challenge. For these ‘R’ treatment groups that were not offered blood meals during the second and third clutch opportunities, no mosquitoes reproduced, indicated by N/A for a not attainable value. The sum of the total eggs laid across the lifespan is denoted as the lifetime reproduction (LTR) and the mean fitness values calculated as the intrinsic rate of increase (*r*). The proportions of mosquitoes that laid eggs at some point over the lifespan were roughly equivalent between treatment groups. Mosquitoes that died from experimental handling or were not compliant with treatment were removed and are not included in the calculations for lifetime reproduction or intrinsic rate of increase

**Fig. 3:**
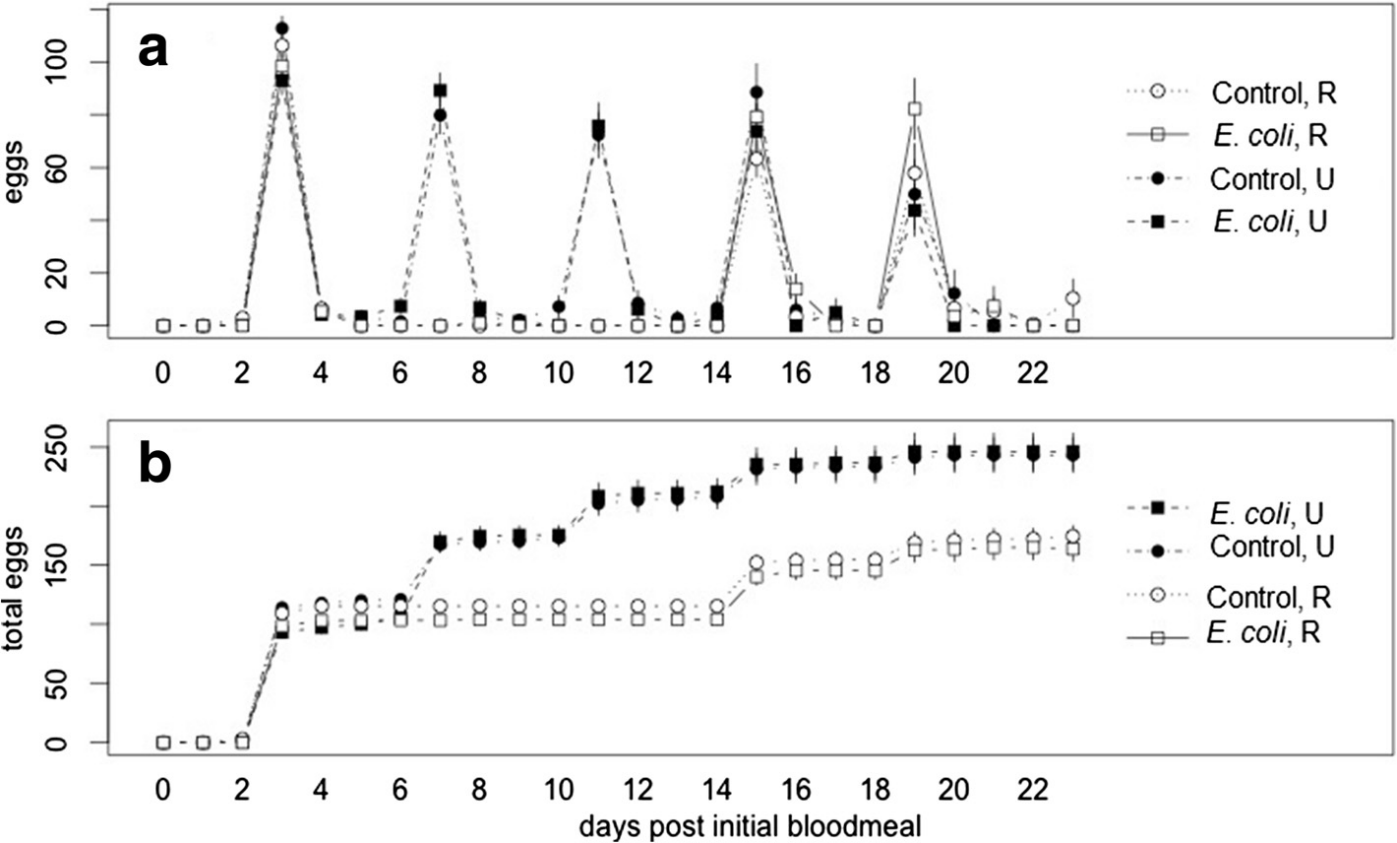
Mosquito reproduction over time. **a** Most mosquitoes laid eggs three days after taking a blood meal, seen by the clustering of peaks on days 3, 7, 11, 15 and 19 after the initial blood meal. Mosquitoes under an immune-challenge produced the same number of eggs and at the same time intervals as unchallenged controls. Only the first clutch laid after an immune-challenge shows a slight reduction in the number of eggs laid, shown by the initially lower number of eggs laid (y-values) of control lines (circles) over the immune-challenged lines (squares). Only mosquitoes that laid at least some eggs over the lifespan are included (total lifetime reproduction > 0). **b** Immune-challenged mosquitoes produced the same cumulative number of eggs over the lifespan as unchallenged controls. Feeding treatment affects lifetime reproduction with mosquitoes on an unaltered ‘U’ feeding regime laying higher cumulative numbers of eggs than those on a restricted ‘R’ feeding regime. Cumulative eggs numbers (y-axis) are the average sums of all eggs laid by mosquitoes in each treatment group up until each day on the x-axis. Mosquitoes that did not lay eggs on a particular day are included in plotted values, so long as the mosquito was alive and reproduced at least once over the lifespan (total lifetime reproduction > 0)

However, for those mosquitoes that reproduced, restricting blood-feeding lowered total lifetime reproduction (Table 1; F_1,452_ = 28.3, p < 0.0001; model selection based on QAIC_c_ values in the Additional file 2: Table S6). Immune-challenge with heat-killed *E. coli* had no impact on lifetime reproduction (Table 1 and Fig. 3; F_1,452_ = 0.1, p > 0.5). If restricted feeding were adaptive during an immune challenge, then for immune challenged mosquitoes, fecundity reductions under restricted blood meal access should be lower or absent compared to the reductions seen in control mosquitoes with a restricted feeding regime. An interaction between blood feeding and immune-challenge would support the host adaptation hypothesis. This was not supported by our analysis. All models accounting for an immune-challenge and feeding regime interaction had less explanatory power than models only accounting for feeding regime (Additional file 2: Table S6, ΔQAIC_c_ >2; specifically, ΔQAIC_c_ =35.5 for the model with the interaction alone, immune-challenge*feeding regime).

Although immune challenge had no impact on total lifetime reproduction, it did affect early fecundity (Table 1) as measured by the size of the initial clutch, with immune-challenged females laying fewer eggs (F_1,415_ = 11.88, p < 0.0007). Although reduced clutch size during an immune challenge was apparent in clutch 1, this effect was not apparent by clutch 2 (Table 1; F_1,116_ = 0.25, p > 0.5).

### The effect of immune-challenge and feeding regime on fitness

Feeding regime had a significant impact on fitness (Table 1; F_1,452_ = 19.28 p < 0.0001; model selection based on ΔAIC_c_ shown in Additional file 2: Table S7), with mosquitoes that fed on an unaltered feeding regime having higher fitness. Immune challenge did not affect fitness (Table 1; F_1,452_ = 2.6, p > 0.1; model selection based on ΔAIC_c_ shown in Additional file 2: Table S7). The predicted effect size of exhibiting an altered feeding behavior, whereby blood meals are not taken until day 12 after the immune-challenge, is a fitness drop of 0.015 (per day), which is an 8 percent fitness reduction. The host adaptation hypothesis predicted this fitness cost to be lower than the fitness cost of the same behavior in controls. The cost was estimated to be the same regardless of challenge.

When mosquitoes on the restricted feeding regime were competed *in silico* against those on an unaltered feeding regime, the time to quasi-loss given an 8 percent fitness difference (*s* = 0.015 per day) was *T* = 309 days. This time period corresponds to the near competitive exclusion of the mosquitoes with altered feeding behavior within roughly 10 mosquito generations.

## Discussion

Illness-induced anorexia is one of the most commonly reported symptoms of infection in host-parasite systems [13–17, 31]. Why such behavior should occur is unclear. Dietary restriction is generally thought to extend lifespan [48–50], and in *Drosophila*, anorexia induced by infection has been shown to increase tolerance to pathogens [17]. In other insects, temporary down regulation of feeding has also been demonstrated to increase the ability of hosts to resist and survive infection [16, 31, 33]. Modulating behavior in the face of infection may be adaptive if these benefits to survival result in overall enhanced fitness. Here we tested whether known restricted blood-feeding behaviors observed in malaria-infected mosquitoes [1–5, 9, 51] provide fitness benefits, suggestive of adaptation. To do this, we presented the first quantification of the impact restricted feeding behaviors have on a composite measure of mosquito fitness and determined that restricted feeding increased survival and decreased lifetime fecundity regardless of immune challenge. When these measurements were incorporated into the composite fitness metric, *r*, we found that restricted feeding behavior negatively impacts mosquito fitness. Contrary to our hypothesis that mosquitoes with restricted feeding behavior during an immune challenge would have reduced fitness costs of delayed blood feeding, mosquitoes exhibiting this phenotype were accruing high fitness costs from delayed reproduction. Why then, do mosquitoes elicit altered feeding behavior during infection?

There are several scenarios that could lead to the apparent maladaptive feeding behaviors induced by infection. One possible scenario is that altered feeding behaviors are adaptive but adaptive benefits were not recapitulated in the lab setting which lacked certain attributes of the mosquito’s natural environment. Females in these experiments fed on anesthetized hosts over short host-seeking scales (<10 cm). While this required orientation, feeding, digestion, egg maturation, and oviposition, the experimental set up lacked other costs associated with feeding, such as host and oviposition site seeking, and host defensive behavior. Assays incorporating additional stressors, such as defensive behaviors, may again reveal costs associated with feeding during the period of behavioral down regulation. Hidden benefits of altered feeding behaviors could also emerge with active parasite infection; however, here we were specifically looking for adaptive benefits to accrue from altered feeding behavior during an immune response alone. Using a high dose of heat-killed *E. coli,* known to elicit a similar immune response and behavioral phenotype as malaria infection [5, 27, 36], allowed us to specifically look at this mosquito response in the absence of parasite dynamics. Although the dose of heat-killed *E. coli* used in these experiments has previously been shown to elicit immune activation [27, 36] and altered feeding responses [5, 27], higher doses of challenge and the larger immune responses they trigger might reveal greater costs of immune challenge and their interaction with feeding. Future work testing less extreme versions of the phenotype, such as delaying one blood meal rather than two, may reveal graduated costs of delayed feeding, or perhaps even fitness benefits under less stressful conditions.

Alternative explanations for why hosts may elicit maladaptive feeding behaviors during infection are that sickness behaviors result from energetic constraints imposed by an immune-response [16, 52], or that these behaviors have evolved via adaptations by the parasite to manipulate host behavior [6]. Untangling possible hypotheses (host adaptation, parasite manipulation or energetic constraints) requires an understanding of the interaction between mosquito and malaria parasite fitness [53]. Previous studies have demonstrated that both active malaria infection [22–24] and mounting a general immune response [25] can decrease fecundity, while effects of infection on mosquito survival are unresolved and seem to vary with host/parasite combination, [20, 54, 55] and dose of challenge [22, 25]. There are few studies incorporating both survival and fecundity over multiple feeding cycles [22] and to our knowledge, no attempts have yet been made to tease apart fitness costs attributable to infection-induced behaviors vs. an infection-induced immune response.

Our restricted blood feeding regimes extended lifespans for mosquitoes, supporting theory that dietary restriction can prolong life [48–50]. Restricting mosquito blood meals during infection, enhanced survival has important implications for disease transmission dynamics. When we compared survivorship over a period equal to the extrinsic incubation period (EIP) of human malaria parasites [44–47], we found that the increased survivorship associated with restricted feeding behavior nearly doubled the number of infectious mosquitoes expected to survive until transmission*.* This further supports predictions that altered behavior could greatly impact vectorial capacity and estimates of transmission potential, or parasite fitness.

While enhanced mosquito survival following dietary restriction is predicted to benefit parasite fitness, our results suggest that the same behavior inflicts heavy fitness costs on the mosquito. Extended survivorship did not compensate for the loss of early reproductive opportunities. As would be expected for insects that are dependent on blood meals to reproduce, restricting blood meals decreases lifetime reproduction. Fewer feeding opportunities translated into fewer reproductive events and lower cumulative numbers of eggs laid over the lifespan. Although compensatory responses in reproductive investment have been demonstrated when re-feeding after food-restriction or stress in other systems [29, 30, 33], there was no evidence that *An. stephensi* females compensated for early fitness losses by increasing reproductive output later in life (Table 1). Our analyses suggest that mosquitoes in restricted treatments would need inordinately large clutches to achieve fitness values comparable to unaltered treatment mosquitoes. As a hypothetical example, mosquitoes laying the same number of clutches after an eight-day delay as experienced by our restricted feeding treatment would need to lay ~1000 x the number of eggs in late clutches to achieve equivalent values for *r*. In the absence of producing larger clutch sizes, these females could also increase clutch frequency. We did not observe any changes in gonotrophic cycle length in our data (Fig. 3) as reported in *Culex* mosquitoes, which shorten their gonotrophic cycle when challenged with a natural malaria pathogen [56].

## Conclusions

Using individual component and composite fitness measures, we found no evidence that altered feeding behavior is adaptive to mosquitoes. In fact, these changes in behavior would be predicted to exacerbate any negative effect of infection on mosquito fitness. Why such behaviors exist remains unclear, though we suggest that the most parsimonious explanation is that they are a consequence of physiological constraints [11, 12] resulting in reproductive opportunity costs. Not all malaria parasite-vector pairs consistently exhibit the behavioral alterations during infection that we explored here [57, 58]. Future exploration of the evolutionary reasons for why some systems and experimental assays exhibit behavioral alterations while others do not, and the role of parasite infection in shaping mosquito blood feeding behaviors and life history are necessary. For mosquitoes known to exhibit altered feeding behaviors during infection, mechanisms and pathways involved in an immune response [26, 27, 52] may limit the ability of mosquitoes to actively host seek during certain periods of infection, resulting in delayed compensatory feeding following these periods [11]. Although parasites could be manipulating the pre-existing relationship between immune-challenge and feeding in mosquitoes to increase transmission (manipulation of compensatory response, [11]), we suggest an alternative: parasites may have evolved developmental cycles in response to physiologically constrained hosts. Parasites reap no fitness gains by reaching the infectious stage prior to female mosquitoes returning to feeding, and thus may benefit more from investment in asexual development in the gut during the period of down-regulated vector feeding behavior. The seemingly coincidental timing of malaria’s extrinsic incubation period, and the timing of the mosquito’s return to feeding following infection, may in fact be a product of parasite evolution in response to host constraints rather than a parasite manipulation of host behavior. If this is the case, and altered feeding behavior is a result of constraints rather than evolutionary adaptation by the mosquito, further understanding of the mechanism of such constraints could lead to the development of novel vector intervention tools.

## Additional files

Additional file 1:
**Additional details of model development, simplification, parameterization, and analysis.** (PDF 374 kb) Additional file 2:
**Tables S1-S7.** This file contains additional data on feeding treatment complience and details of model outputs. (DOCX 503 kb)
