# Supplementary material for: Fitness consequences of altered feeding behavior in immune-challenged mosquitoes
Source: Parasit Vectors. 2016 Feb 29;9:113. doi: 10.1186/s13071-016-1392-x (PMC4772315; doi:10.1186/s13071-016-1392-x)
Supplement: Additional file 1: — Additional details of model development, simplification, parameterization, and analysis. (PDF 374 kb) [file 13071_2016_1392_MOESM1_ESM.pdf]

1 Model development

Here we develop a population-scale model that uses individual-scale life table information to estimate a fitness measure for each individual in the experiment. In essence, the model gives the population growth that would result if a population was comprised of individuals with identical life histories based on the empirical life table of a single individual. While the model is developed for mosquitoes in this experiment, the approach is general to organisms with distinct stages.

The experiments exposed adult mosquitoes to different treatments and recorded the day of death and the number of eggs laid each day. Let $E(t)$, $L(t)$ and $A_i(t)$ denote the density of eggs, larvae and adults respectively at time t. For adults, i denotes the time since pupal eclosion. We assume that all individuals in a stage share the same development, birth and mortality rates. To match the experimental protocol, we assume that i is an integer number of days since pupal eclosion. Specifically, $i \in \{0, 1, \ldots, \alpha\}$ where α is the last day the individual was alive. The birth rate b_i differs depending on the age since pupal eclosion as observed in the experiments. To account for mortality, the model assumes that individuals in the egg and larval stages have constant daily mortality rates, but that adult mortality occurs exactly as observed in the data. This is done by introducing a recruitment term out of the adult class at a time delay given by the observed longevity for that individual.

The population model is

$$\frac{dE(t)}{dt} = \sum_{i=0}^{\alpha} b_i A_i(t) - \sum_{i=0}^{\alpha} b_i A_i(t_1) S_E - \delta_E E \tag{1}$$

$$\frac{dL(t)}{dt} = \sum_{i=0}^{\alpha} b_i A_i(t_1) S_E - \sum_{i=0}^{\alpha} b_i A_i(t_2) S_E S_L - \delta_L L \tag{2}$$

$$\frac{dA_j(t)}{dt} = \sum_{i=0}^{\alpha} b_i \left(A_i(t_2 - j) - A_i(t_2 - j - 1)\right) S_E S_L \tag{3}$$

$$S_E = \exp(-\delta_E \tau_E) \tag{4}$$

$$S_L = \exp(-\delta_L \tau_L) \tag{5}$$

$$t_1 = t - \tau_E \tag{6}$$

$$t_2 = t - \tau_E - \tau_L \tag{7}$$

where τ_E and τ_L are the egg and larvae stage durations, δ_E and δ_L are the stage-specific mortality rates, and S_E and S_L are through stage survivorship's. The second term of (3) is recruitment about of the adult class, which describes the death of adults at an exact age since pupal eclosion of α. The index i is used to integrate reproduction over all adult ages, and the index j denotes the dynamics of adult densities at each age. The full system has dimension $\alpha + 3$.

2 Model simplification

Since the adult stage equation does not depend on egg or larval densities, we can model the population growth using just the adult equations as

$$\frac{dA_j(t)}{dt} = S\left(\sum_{i=0}^{\alpha} b_i\left(A_i(t_2 - j) - A_i(t_2 - j - 1)\right)\right) \tag{8}$$
$$S = \exp(-\delta_E\tau_E - \delta_L\tau_L) \tag{9}$$
$$t_2 = t - \tau_E - \tau_L \tag{10}$$

for $j \in \{0, \ldots, \alpha\}$. Since the model is density independent, adult densities will asymptotically undergo exponential growth or decay. While it is possible to write the corresponding characteristic equation for this model, there are no analytical methods to solve for the maximum growth rate. To simplify the process of estimating the growth rate, we write the discrete-time approximation to the model as

$$\mathbf{n}_{t+1} = \mathbf{L}\mathbf{n}_t \tag{11}$$

where $\mathbf{n}_t$ is a vector of the number of mosquitoes in a particular age class at time t, and L is a Leslie matrix given by

$$\mathbf{L} = \begin{bmatrix} 0 & 0 & \ldots & 0 & 0 & \ldots & b_0 & b_1 & \ldots & b_{\alpha-1} & b_\alpha \\ e^{-\delta_E} & 0 & \ldots & 0 & 0 & \ldots & 0 & 0 & \ldots & 0 & 0 \\ 0 & e^{-\delta_E} & \ldots & 0 & 0 & \ldots & 0 & 0 & \ldots & 0 & 0 \\ \vdots & \vdots & \ddots & \vdots & \vdots & \vdots & \vdots & \vdots & \ddots & \vdots & \vdots \\ 0 & 0 & \ldots & e^{-\delta_L} & 0 & \ldots & 0 & 0 & \ldots & 0 & 0 \\ 0 & 0 & \ldots & 0 & e^{-\delta_L} & \ldots & 0 & 0 & \ldots & 0 & 0 \\ \vdots & \vdots & \ddots & \vdots & \vdots & \ddots & \vdots & \vdots & \ddots & \vdots & \vdots \\ 0 & 0 & \ldots & 0 & 0 & \ldots & 1 & 0 & \ldots & 0 & 0 \\ 0 & 0 & \ldots & 0 & 0 & \ldots & 0 & 1 & \ldots & 0 & 0 \\ \vdots & \vdots & \ddots & \vdots & \vdots & \ddots & \vdots & \vdots & \ddots & \vdots & \vdots \\ 0 & 0 & \ldots & 0 & 0 & \ldots & 0 & 0 & \ldots & 1 & 0 \end{bmatrix} \tag{12}$$

where the age classes start from the first egg stage and go until the observed day of adult death ($\tau_E+\tau_L+\alpha$). Note that, as in the full model, adult mortality is incorporated explicitly through senescence through the dimension of the transition matrix ($\mathbf{L}$).

3 Model parameterization from life table data

The experimental protocol was to work with adult mosquitoes immediately after pupal eclosion. As a result, development times and mortality rates of the egg and larval stages will be the same for all treatments. We use egg and larval development times of $\tau_E = 1\ d$ and $\tau_L = 12\ d$ respectively, which are typical for this lab population at the temperature and humidity conditions of the experiments (1). To estimate egg and larval mortality rates, we use the observation that roughly 70% of eggs hatch (Moller-Jacobs, unpublished data), and 75% of the individuals survive from egg eclosion to pupal eclosion (1), which corresponds to egg and larval mortality rates of $\delta_E = -\ln(0.7)/\tau_E = 0.356\ d^{-1}$ and $\delta_L = -\ln(0.75)/\tau_L = 0.024\ d^{-1}$ respectively. The remaining parameters are the age of adult death (α) and daily birth rate (b_i), which are obtained directly from the life-table data for each individual.

4 Model analysis and evaluation of assumptions

The life-table data for each individual leads to a unique parameterization of the population model, and therefore a unique value of the asymptotic population growth rate. Recognizing that the inference is constrained to these laboratory conditions under an assumption of unlimited resources, the calculated population growth provides an estimate of fitness that properly combines the role of fecundity and survivorship. The asymptotic growth rate for the population model in eqn (11) is given by the right leading eigenvalue using the *eigen* function in the R statistical environment (2). Fitness for the full continuous-time model was estimated through time simulations using the *PBSddesolve* library (3) in the R statistical environment (2).

For each individual, we calculated a fitness estimate using the full continuous-time model in eqns (1)-(7) and the discrete-time approximation in eqn (11). The estimates of fitness from both models were in excellent agreement (Figure 1), which indicates that the approximation is valid for these experiments. We used the full model when reporting statistical results, and for computational speed used the discrete-time model to explore the robustness of our conclusions to the values of egg and larval development and mortality (i.e., τ_E, τ_L, δ_E, δ_L). We explored changes from 10% to 500% for each parameter and found the statistical results are robust to variation in these parameters (Figure 2), but the effect size increases with increasing mortality rates and development times (Figure 3).

References

[1] Moller-Jacobs L, Murdock CC, Thomas MB (2014). Capacity of mosquitoes to transmit malaria depends on larval environment. Parasites & Vectors. 7(593): doi:10.1186/s13071-014-0593-4.

[2] R Core Team (2014). R: A language and environment for statistical computing. R Foundation for Statistical Computing, Vienna, Austria. URL http://www.R-project.org/.

[3] Couture-Beil A, Schnute JT, Haigh R, Boers N, Wood SN, Cairns BJ (2014). PBSddesolve: Solver for Delay Differential Equations. R package version 1.11.29. http://CRAN.R-project.org/package=PBSddesolve.

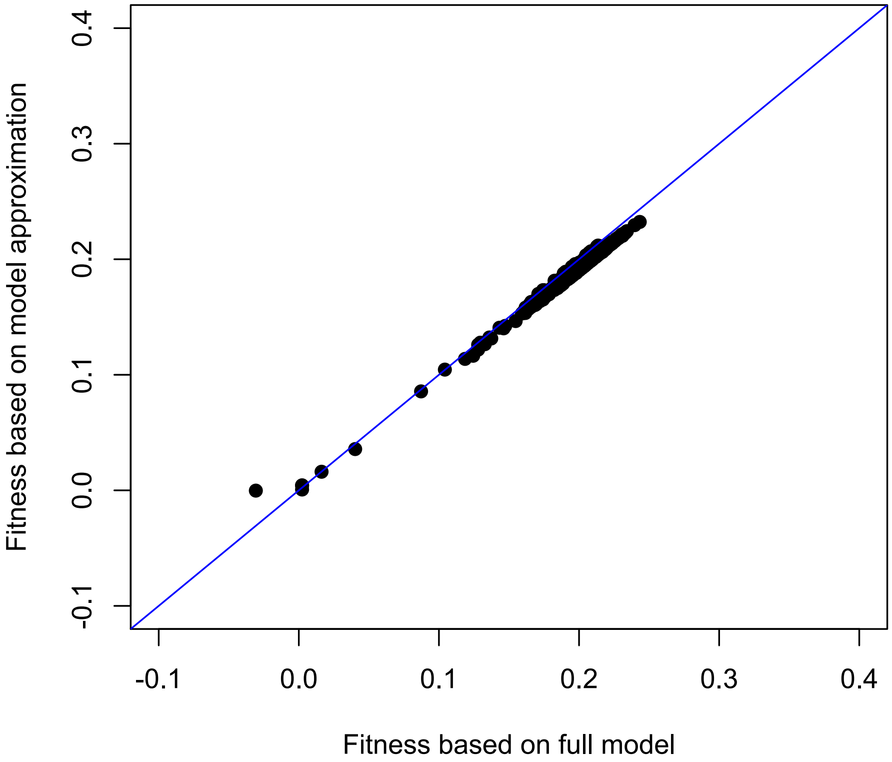

Fig. 1. Fitness estimates based on the full model in eqns (1)-(7) compared to fitness estimates based on the discrete-time approximation in eqn (11). The one-to-one line is shown in blue.

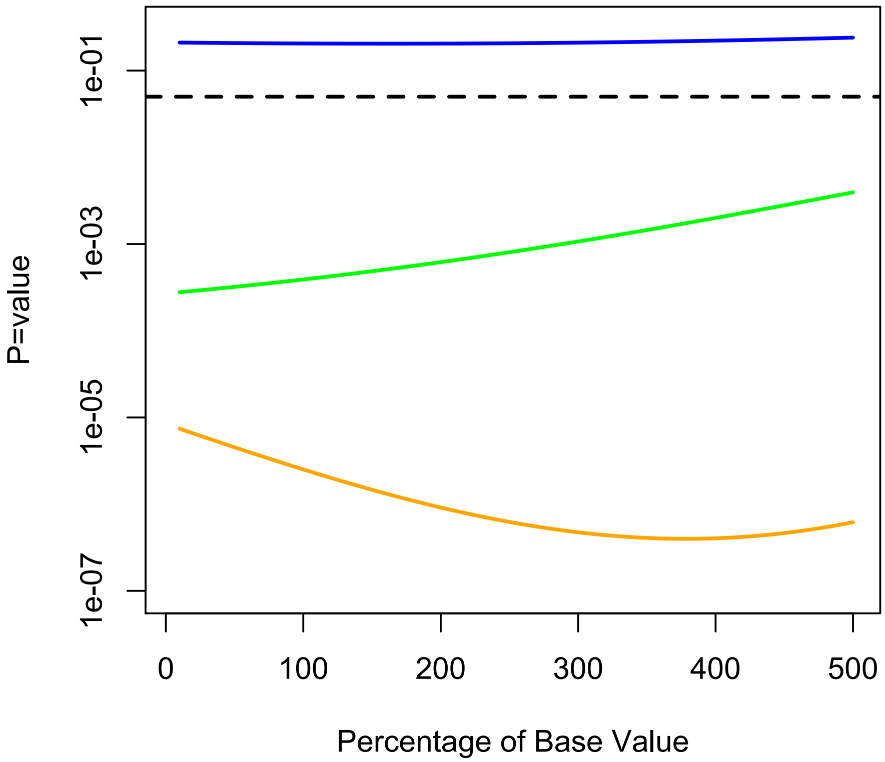

Fig. 2. Change in the p-value of the statistical tests for treatment effects on fitness as a function of changes in the egg and larval parameter values for replicate 2. The interaction term (blue line), bloodmeal treatment (orange line) and immune challenge treatment (green line) are shown along with the chosen critical p-value of $p = 0.05$ (dashed line).

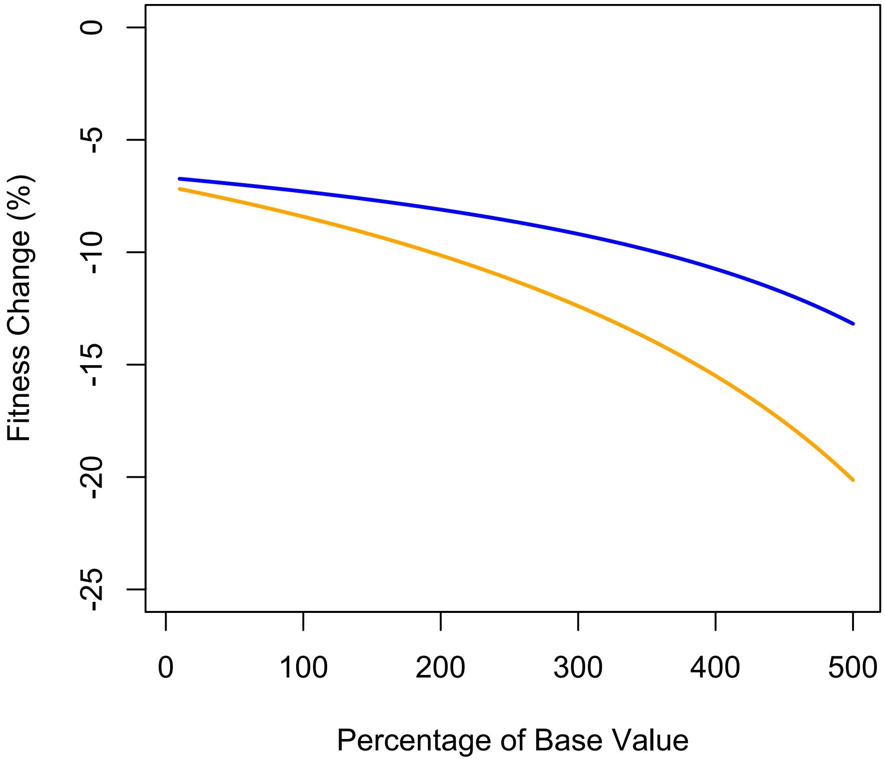

Fig. 3. Percent change in fitness (relative to control) as a function of changes in the egg and larval parameter values for replicate 2. The effect of skipping a blood meal is shown in orange and the effect of immune challenge is shown in blue.
