# Supplementary material for: Fitness consequences of altered feeding behavior in immune-challenged mosquitoes
Source: Parasit Vectors. 2016 Feb 29;9:113. doi: 10.1186/s13071-016-1392-x (PMC4772315; doi:10.1186/s13071-016-1392-x)
Supplement: Additional file 2: — Tables S1-S7. This file contains additional data on feeding treatment complience and details of model outputs. (DOCX 503 kb) [file 13071_2016_1392_MOESM2_ESM.docx]

**Supplementary Information**
**Table S1.** Feeding treatment compliance.

| Replicate 1 | Treatment | Day 4 | Day 8 | Day 12 | Day 16 |
| --- | --- | --- | --- | --- | --- |
|  | ***E. coli* immune-challenged, UR** | **0 (0:45)** | **0 (0:29)** | **0 (0:17)** | **0 (0:8)** |
|  | *E. coli* immune-challenged, R | No feed | No feed | 0 (0:36) | 0 (0:16) |
|  | **Control, UR** | **0 (0:60)** | **0 (0:37)** | **0 (0:25)** | **0 (0:11)** |
|  | Control, R | No feed | No feed | 0.02 (1:48) | 0.04 (1:25) |
| Replicate 2 | ***E. coli* immune-challenged, UR** | **0.04 (3:74)** | **0.09 (5:51)** | **0.06 (2:32)** | **0.13 (3:21)** |
|  | *E. coli* immune-challenged, R | No feed | No feed | 0.09 (6:64) | 0.07 (3:40) |
|  | **Control, UR** | **0.18 (13:60)*** | **0.06 (3:45)** | **0.15 (5:28)** | **0.16 (4:21)** |
|  | Control, R | No feed | No feed | 0.13 (7:48) | 0 (0:32) |
|  | **Sham, UR** | **0.08 (5:58)** | **0.06 (3:48)** | **0.09 (3:29)** | **0.05 (1:20)** |
|  | Sham, R | No feed | No feed | 0.06 (3:47) | 0.03 (1:29) |

Not all mosquitoes fed when offered a blood meal. Non-compliers were censored from the experiment on the day of non-compliance. Days when mosquitoes were offered blood meals and the proportion of mosquitoes in each treatment that failed to comply on that day are listed in columns. In parentheses is the ratio of the number of mosquitoes that did not feed: number of mosquitoes that fed. ‘UR’ indicates the unrestricted blood feeding regime and ‘R’ indicates restricted. Challenged ‘UR’ treatments would have lower compliance than ‘UR’ controls if the feeding behavior extended to short-range host-seeking, ‘UR’ treatments are highlighted to facilitate this comparison. Compliance was higher in Replicate 1. Asterix (*) indicates the only treatment that had significantly higher non-compliance.

**Table S2.** Evaluation of sham treatment (physical damage, no physical damage) for proportion reproducing (PR). Best fit model highlighted in bold, models ranked from best to least fit.

| Model | qAIC | delta qAIC | Degrees of Freedom |
| --- | --- | --- | --- |
| **PR~1 (null model)** | **427.2** | **0** | **2** |
| PR~physical damage | 429.2 | 2 | 3 |

**Table S3.** Evaluation of sham treatment (physical damage, no physical damage) for lifetime reproduction. Best fit model highlighted in bold, models ranked from best to least fit.

| Model | qAIC | delta qAIC | Degrees of Freedom |
| --- | --- | --- | --- |
| **LTR~1 (null model)** | **190.3** | **0** | **2** |
| LTR~physical damage | 192.3 | 2 | 3 |

**Table S4.** Evaluation of sham treatment (sham damage, no damage) on fitness (r). Best fit model highlighted in bold, models ranked from best to least fit.

| Model | AIC | delta AIC | Degrees of Freedom |
| --- | --- | --- | --- |
| **Fitness~1 (null model)** | **-85652.7** | **0** | **2** |
| Fitness~physical damage | -85657.8 | 5.1 | 3 |

**Table S5.** Evaluation of immune challenge and feeding treatments, and experimental replicate on proportion reproducing (PR). Best fit model highlighted in bold, models ranked from best to least fit.

| Model | qAIC | delta qAIC | Degrees of Freedom |
| --- | --- | --- | --- |
| **PR~immune-challenge*replicate** | **892.2** | **0** | **5** |
| PR~immune-challenge*replicate+feeding regime | 893.2 | 1 | 6 |
| PR~immune-challenge*feeding regime*replicate | 894.9 | 2.7 | 9 |
| PR~1 (null model) | 896.0 | 3.8 | 2 |
| PR~immune-challenge*feeding regime | 896.6 | 4.4 | 5 |
| PR~feeding regime | 897.2 | 5 | 3 |
| PR~immune-challenge | 897.3 | 5.1 | 3 |
| PR~immune-challenge+feeding regime | 898.4 | 6.2 | 4 |
| PR~immune-challenge*feeding regime+replicate | 898.5 | 6.3 | 6 |
| PR~feeding regime+replicate | 899.2 | 7 | 4 |
| PR~immune-challenge+replicate | 899.2 | 7 | 4 |
| PR~immune-challenge+feeding regime+replicate | 900.4 | 8.2 | 5 |
| PR~F*replicate | 900.7 | 8.5 | 5 |
| PR~immune-challenge+feeding regime*replicate | 901.9 | 9.7 | 6 |

**Table S6.** Evaluation of immune-challenge and feeding regime treatments, and experimental replicates 1 & 2 on total lifetime reproduction (LTR), measured as the sum of all eggs produced per female. Best fit model highlighted in bold, models ranked from best to least fit.

| Model | qAIC | delta qAIC | Degrees of Freedom |
| --- | --- | --- | --- |
| **LTR~feeding regime + replicate** | **465.7** | **0** | **4** |
| LTR~immune-challenge+feeding regime+ replicate | 466.6 | 0.9 | 5 |
| LTR~feeding regime* replicate | 467.2 | 1.5 | 5 |
| LTR~immune-challenge+feeding regime+replicate | 468.0 | 2.3 | 6 |
| LTR~immune-challenge*replicate+feeding regime | 468.1 | 2.4 | 6 |
| LTR~immune-challenge*feeding regime+replicate | 468.5 | 2.8 | 6 |
| LTR~immune-challenge*feeding regime*replicate | 471.3 | 5.6 | 9 |
| LTR~feeding regime | 497.6 | 31.9 | 3 |
| LTR~immune-challenge+feeding regime | 499.4 | 33.7 | 4 |
| LTR~immune-challenge+replicate | 499.7 | 34.0 | 4 |
| LTR~immune-challenge*feeding regime | 501.2 | 35.5 | 5 |
| LTR~immune-challenge*replicate | 501.5 | 35.8 | 5 |
| LTR~1 (null model) | 531.7 | 66.0 | 2 |
| LTR~immune-challenge | 532.9 | 67.2 | 3 |

**Table S7.** Evaluation of immune challenge and feeding treatments, and experimental replicate on fitness (r). Best fit model highlighted in bold, models ranked from best to least fit.

| Model | AIC | delta AIC | Degrees of Freedom |
| --- | --- | --- | --- |
| **Fitness~feeding regime** | **-1259.2** | **0** | **3** |
| Fitness~immune-challenge+feeding regime | -1259.0 | 0.2 | 4 |
| Fitness~immune-challenge*feeding regime | -1257.4 | 1.9 | 5 |
| Fitness~feeding regime+replicate | -1257.3 | 2.0 | 4 |
| Fitness~immune-challenge+feeding regime+replicate | -1257.0 | 2.2 | 5 |
| Fitness~immune-challenge*replicate+feeding regime | -1256.3 | 2.9 | 6 |
| Fitness~feeding regime*replicate | -1256.2 | 3.0 | 5 |
| Fitness~immune-challenge+feeding regime*replicate | -1256.0 | 3.2 | 6 |
| Fitness~immune-challenge*feeding regime+replicate | -1255.4 | 3.8 | 6 |
| Fitness~immune-challenge | -1253.9 | 5.3 | 3 |
| Fitness~1 (null model) | -1253.6 | 5.6 | 2 |
| Fitness~immune-challenge+replicate | -1251.9 | 7.3 | 4 |
| Fitness~immune-challenge*feeding regime*replicate | -1251.6 | 7.7 | 9 |
| Fitness~immune-challenge*replicate | -1251 | 8.2 | 5 |
